# Sweet Immunity: The Effect of Exogenous Fructans on the Susceptibility of Apple (*Malus × domestica* Borkh.) to *Venturia* *inaequalis*

**DOI:** 10.3390/ijms21165885

**Published:** 2020-08-16

**Authors:** Anze Svara, Łukasz Paweł Tarkowski, Henry Christopher Janse van Rensburg, Evelien Deleye, Jarl Vaerten, Nico De Storme, Wannes Keulemans, Wim Van den Ende

**Affiliations:** 1 Laboratory for Fruit Breeding and Biotechnology, Division of Crop Biotechnics, KU Leuven, 3001 Leuven, Belgium; anze.svara@kuleuven.be (A.S.); evelien.deleye@hotmail.com (E.D.); jarl.vaerten@hotmail.com (J.V.); nico.destorme@kuleuven.be (N.D.S.); wannes.keulemans@kuleuven.be (W.K.); 2 Seed Metabolism and Stress Team, INRAE Angers, Institut de Recherche en Horticulture et Semences, Bâtiment A, CEDEX, 49071 Beaucouzé, France; lukasz.tarkowski@inrae.fr; 3 Lab of Molecular Plant Biology, KU Leuven, 3001 Leuven, Belgium; henry.jansevanrensburg@kuleuven.be

**Keywords:** apple, scab, fructans, *Malus*, *Venturia*, leaves, priming, fungicides, biocontrol

## Abstract

There is an urgent need for novel, efficient and environmentally friendly strategies to control apple scab (*Venturia inaequalis*), for the purpose of reducing overall pesticide use. Fructans are recently emerging as promising “priming” compounds, standing out for their safety and low production costs. The objective of this work was to test a fructan-triggered defense in the leaves of apple seedlings. It was demonstrated that exogenous leaf spraying can reduce the development of apple scab disease symptoms. When evaluated macroscopically and by *V. inaequalis*-specific qPCR, levan-treated leaves showed a significant reduction of sporulation and *V. inaequalis* DNA in comparison to mock- and inulin-treated leaves, comparable to the levels in fosetyl-aluminum-treated leaves. Furthermore, we observed a significant reduction of *in vitro* mycelial growth of *V. inaequalis* on plates supplemented with levans when compared to controls, indicating a direct inhibition of fungal growth. Variations in endogenous sugar contents in the leaves were followed during priming and subsequent infection, revealing complex dynamics as a function of time and leaf ontogeny. Our data are discussed in view of the present theories on sugar signaling and fructan-based immunity, identifying areas for future research and highlighting the potential use of fructans in apple scab management in orchards.

## 1. Introduction

Domesticated apple (*Malus × domestica* Borkh.) is the most important fruit crop in the temperate climates, with a total annual production of 89.3 million metric tons [1]. It is continuously threatened by numerous diseases, among which the most dangerous is apple scab caused by the hemi-biotrophic fungal pathogen *Venturia inaequalis* [2,3]. Due to its aggressive epidemiology and dynamic life cycle divided into a saprophytic and biotrophic phase, effective scab control during its biotrophic phase is based on intensive fungicide spraying schemes, with an average of 18–25 applications for a single growing season [4,5,6,7,8]. This strategy has severe drawbacks, such as high costs, negative environmental impact and emergence of fungicide-resistant *V. inaequalis* strains [3,7,9,10]. To reduce the dependence of farmers on expensive fungicide applications, alternatives can be taken into consideration, including the breeding of scab-resistant varieties. Introgression of disease resistance genes derived from wild *Malus* species into *M.* × *domestica* germplasm gained considerable attention and led to a number of scab-resistant cultivars [5,11,12]. However, such breeding processes are time-consuming and require several pseudo-backcrosses, where not only one parent line but different parents from the same species are used to eliminate undesirable traits [13,14]. Thus, despite the fact that several loci associated with scab resistance were identified, the number of resistant cultivars is limited, and today, the great majority of commercially relevant cultivars remains susceptible to scab [13,15,16].

Disadvantages associated with breeding and chemical control techniques paved the way to the use of more affordable and sustainable approaches, widely termed as biological control [17,18]. Among them, promising results were obtained by the exogenous application of sustainable compounds that are capable of eliciting (induction of defense system by small molecules that are distinct from endogenous plant defense signaling molecules) a stronger and/or faster defense activation in response to a subsequent (a)biotic stressor [19,20,21]. Such processes are generally termed as “priming” [22]. A priming compound is expected to boost resistance without major drawbacks in plant fitness, the reproductive success of a plant [22]. However, research showed that a minor decrease in fitness is common in primed plants, but such a disadvantage is largely compensated by the improved capacity to cope with a stressful environment [23,24]. Priming of plant defenses with natural compounds offers an alternative or supporting platform to current practices, especially under conditions of high disease pressure, resulting in a reduced number of fungicide treatments [25]. This is particularly the case when there is a high host susceptibility, such as in the cultivated apple-*Venturia* interactions.

To date, a number of compounds belonging to different chemical classes have been tested as priming agents, including amino acids such as β- and γ-aminobutyric acid (BABA and GABA, respectively) [26,27], organic acids [28] and inorganic compounds such as silicon and fosetyl-aluminum (F-Al) [29,30]. The latter is a synthetic compound that shows a high effectivity towards apple scab disease development. Its mode of action is dual: on the one hand, it inhibits fungal germination (fungicide action); on the other hand, it is believed to prime plant defenses [31,32]. On top of that, the use of carbohydrates as stimulators of plant defenses is being supported by a steadily growing body of evidence [33,34,35]. Carbohydrates are among the cheapest, most abundantly available and sustainable potential priming compound classes, and besides being central in the primary metabolism, they also exert critical signaling roles in various plant developmental processes and stress responses [36,37,38,39]. It is well-established that sugars contribute to plant immune mechanisms [40,41,42], and these observations led to the definition of the term “sweet immunity”, referring to the involvement of sugar metabolism and signaling in boosting plant defense responses [43]. A number of structural carbohydrates, such as pectin-derived oligogalacturonides (OGs), cellobiose (disaccharide of cellulose), β-1,3 glucans, chitin (*n*-acetyl glucosamine polymer) and its deacetylated derivative chitosan, were already shown to effectively increase plant resistance in different plant-pathogen interactions via exogenous application [20,33,34,35,44,45]. Some of them may function as microbe- or damage-associated molecular patterns (MAMPs or DAMPs, respectively) in the pathogen recognition process. 

**Figure 1:**
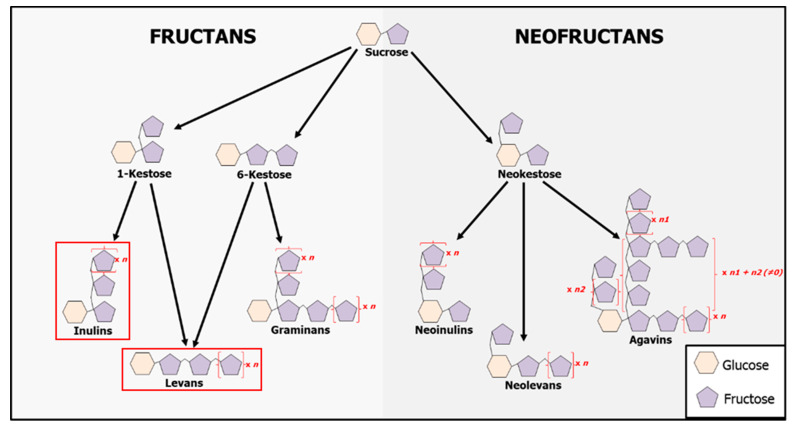
Schematic illustration of structural variants of fructans found in plants (adapted from [53]). Fructans are produced and stored in vacuoles. The fructan variants used in this work are highlighted by a red box. Their synthesis is carried out by enzymes named fructosyltransferases, whereas their degradation is done by fructan exohydrolases [46].

Besides structural carbohydrates, the involvement of metabolic sugars in biotic stress resistance is emerging [41]. One of the most important members of this class is fructans, fructose-based oligo- and polysaccharides synthesized by elongating a sucrose (Suc) molecule with fructose (Fru) moieties [46]. These carbohydrates occur in approximately 15% of higher plants, including some economically important families (Poaceae and Asteraceae) [47]. Fructan accumulation is frequently correlated with an increased tolerance to abiotic stresses such as cold and water deficits [48,49,50,51]. Fructans are subdivided into different classes, mainly based on the linkage type between adjacent Fru moieties. Six classes of plant fructans can be discerned, including inulins (β(2-1) linkages), levans (β(2-6) linkage), graminans (mixed linkages) and different neokestose-based fructans, further divided into neoinulins, neolevans and agavins [46,52] (Figure 1). 

The exogenous leaf spraying of inulin-type fructans from burdock (*Arctium lappa*) roots boosted disease resistance against fungi and viruses in lettuce, tomato and tobacco [53,54,55]. These studies suggested that fructans may be potentially useful compounds for wider disease control both in pre- and post-harvest contexts and led to the hypothesis that fructans may be perceived as MAMPs and/or DAMPs in plants [56]. Thus, fructans may be interesting defense elicitors, especially in terms of sustainability and affordability. To date, no reports have been published on the use of exogenous leaf spraying of fructans in the context of the apple-*Venturia* pathosystem, despite promising results in other plant-pathogen interactions and applications on post-harvest apple fruits [57]. On the other hand, several other compounds have been studied as defense elicitors to improve disease management and resistance against apple scab. Examples include potassium phosphate, potassium carbonate, salicylic acid (SA) and its derivatives, silicon and chitosan [58,59,60,61]. However, despite their successful defense enhancement, various side/mixed effects, such as difficulties with formulation and environmental effects (e.g., ground water contamination), were observed [62,63].

Here, we have explored the possibility of using fructans as a potential environmental-friendly and cost-efficient defense elicitor in the youngest leaves of apple seedlings challenged with *V. inaequalis*. We discuss our findings based on the current views on fructan-mediated immunity. 

## 2. Results

### 2.1. Levan Application Significantly Decreases V. inaequalis Sporulation on Leaves of Apple Seedlings

**Figure 2:**
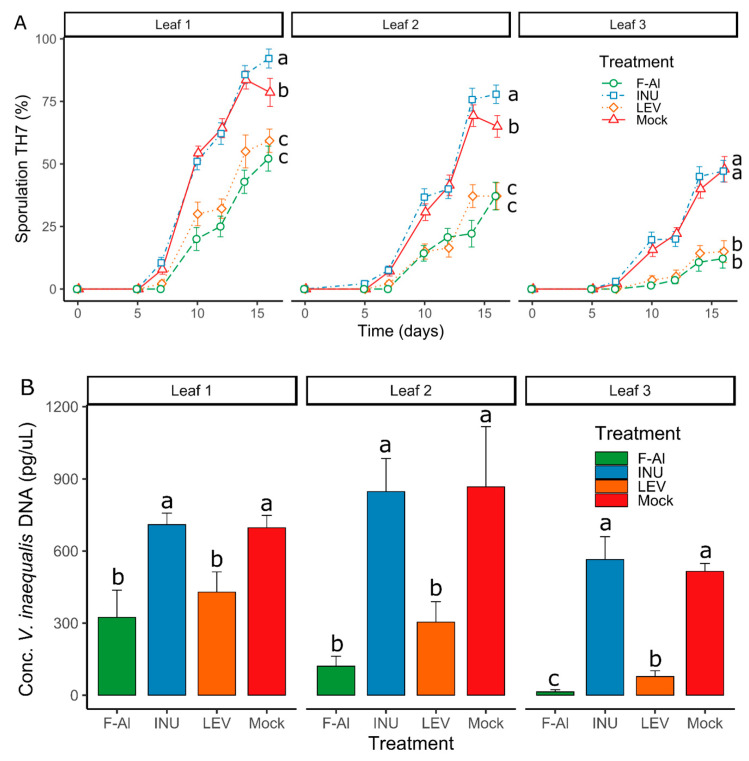
(**A**) Means of TH7 sporulation symptoms of *Venturia inaequalis* on apple leaves sprayed with different elicitors. F-Al = fosetyl-Al 1.25 g L^−1^, INU = inulin 1 g L^−1^, LEV = levan 1 g L^−1^, Mock = water-treated and TH7 = Townsend-Heuberger with the number of symptom-showing classes (*n* = 20 ± SE). Statistical significance at 16 days post-inoculation between treatments is indicated by different letters (*p* < 0.05). (**B**) Means of DNA concentrations of *V. inaequalis* on all 3 leaves at 14 days post-inoculation (dpi). F-Al = fosetyl-Al, INU = inulin, LEV = levan and Mock = water-treated (*n* = 6 ± SE). Statistical significance between treatments is indicated by different letters (*p* < 0.05). The experiment was repeated 3 times with consistent results.

**Figure 3:**
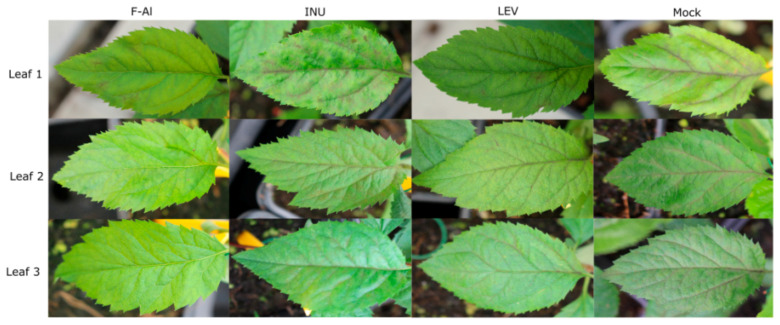
Representative pictures of leaves infected with *Venturia inaequalis* after treatment with fructans and fosetyl-Al at 14 dpi. F-Al = fosetyl-Al 1.25 g L^−1^, INU = inulin 1 g L^−1^, LEV = levan 1 g L^−1^ and Mock = water-treated.

Firstly, we assessed the defense-enhancing potential of the different fructans on the youngest three leaves of apple seedlings after *V. inaequalis* inoculation. Results show that levans significantly decrease sporulation compared to the mock in all three leaves tested at 16 days post-inoculation (dpi) (Figure 2A). Notably, levan-treated leaves showed a significant reduction in sporulation already from 7 dpi onward, following a similar sporulation profile as F-Al-treated leaves (Figure 2A and Figure 3). On the other hand, inulin-type fructans did not provide protection in any of the leaves tested (Figure 2A). Inulin-sprayed leaves displayed even higher *V. inaequalis* sporulation on leaf 1 and 2 at 16 dpi compared to the mock (Figure 2A). *Venturia inaequalis* DNA quantification data supported the sporulation symptoms data, with samples treated with F-Al and levans showing significantly decreased *V. inaequalis* DNA concentrations in the leaves at 14 dpi compared to the mock and inulin treatments (Figure 2B). In particular, leaf 3 (older leaf) seems to be the most responsive to levan treatment (reduction in *V. inaequalis* DNA in the levan-treated leaves in comparison to the mock-treated ones was 38.48%, 64.95% and 84.94% in the leaves 1, 2 and 3, respectively) and showed the highest ontogenic resistance to *V. inaequalis* infection. The quantification of chlorosis and necrosis symptoms resulted in smaller differences between the treatments or even their absence. However, an increase in necrosis for all the treatments in comparison to the levan treatment and an increase in chlorosis for the inulin treatment was observed for all the leaves tested (Figure S1).

### 2.2. Infection, but not Priming, Induces Suc Accumulation

**Figure 4:**
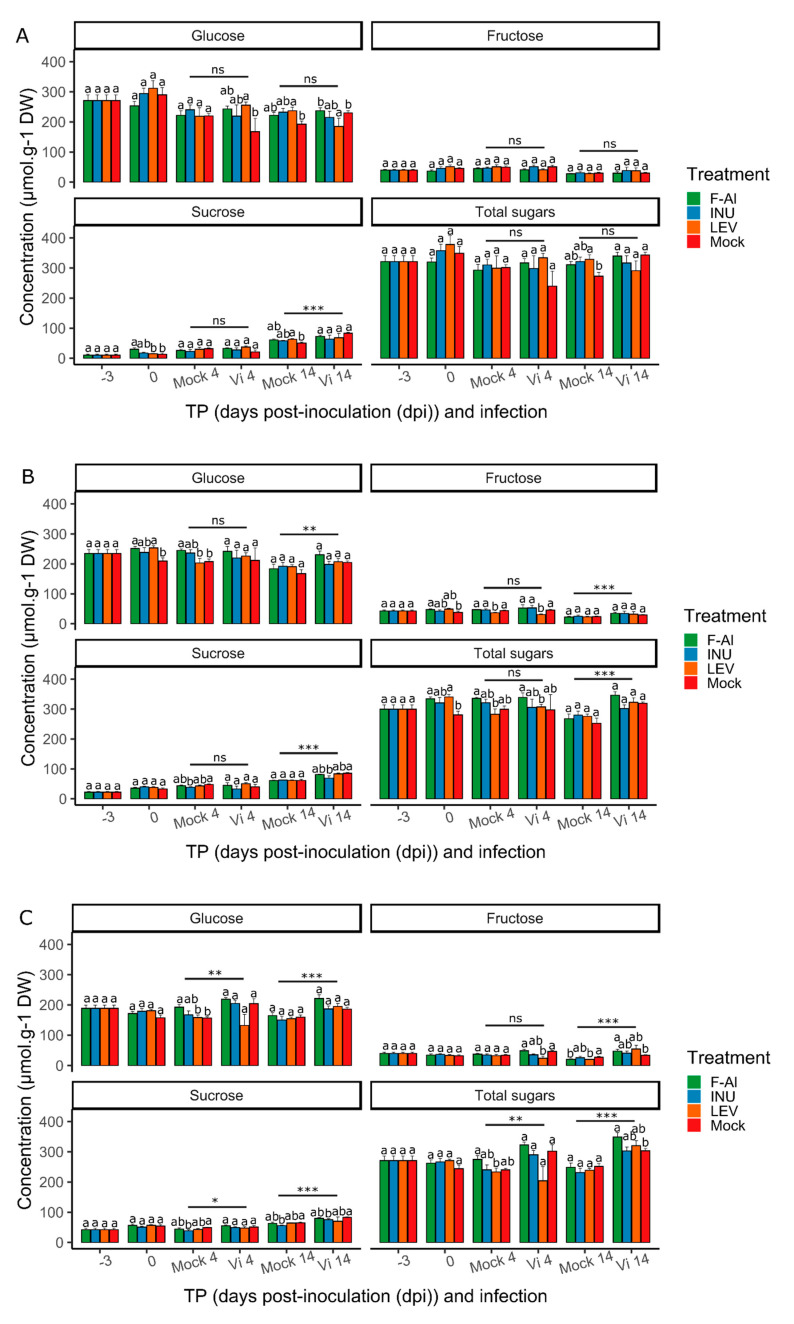
Soluble sugar concentrations (in µmoles/g dry weight (DW)) in leaves infected with *Venturia inaequalis* as a function of time (dpi). F-Al = fosetyl-Al, INU = inulin, LEV = levan, Mock = water-treated, TP = time point and dpi = days post-inoculation. On the X-axis, Mock represents leaves inoculated with the mock solution, while Vi means that leaves were inoculated with spores of *V. inaequalis*. Total sugars are defined as the sum of the glucose, fructose and sucrose concentrations. (**A**) Sugar concentrations in leaf 1. (**B**) Sugar concentrations in leaf 2. (**C**) Sugar concentrations in leaf 3 (*n* = 6 ± SE). Letters indicate statistical differences between treatments at a certain TP (*p* < 0.05). Asterisks indicate treatment-independent differences between Vi and mock. This experiment was repeated 2 times with consistent results.

To investigate whether exogenous levan treatments lead to changes in soluble sugar dynamics, we analyzed the concentrations of glucose (Glc), Fru and Suc in leaves 1, 2 and 3 at different time points (TPs): the time of priming (−3 dpi), right before inoculation (0 dpi) and after inoculation (4 and 14 dpi). We also included a mock-inoculated control for 4 and 14 dpi to assess the impact of the *V. inaequalis* infection on the soluble sugar dynamics (Figure 4). Overall, the data demonstrate that the treatments had little effect on the soluble sugar concentrations, with some small but significant exceptions, such as a levan effect on Fru levels at 4 dpi in leaves 2 and 3 (Figure 4B,C) and a F-Al effect on Suc in leaf 1 at 0 dpi (Figure 4A). Notably, the progression of the *V. inaequalis* infection did not influence the sugar concentrations to a great extent, with only small differences between the mock- and *V. inaequalis*-inoculated samples at 4 and 14 dpi for all three leaves (Figure 4). However, irrespective of the treatments, a significant increase in the measured sugar concentrations was detected in the infected samples as compared to the mock-inoculated samples. The increase in Glc, Suc and total sugars at 4 dpi was detected only in leaf 3 (Figure 4C) and for all the measured sugars at 14 dpi in leaves 2 and 3 (Figure 4B,C). In leaf 1, only Suc was increased at 14 dpi (Figure 4A). In addition to the response to the infection with *V. inaequalis*, Suc concentrations were higher in older leaves at TPs −3, 0 and 4 dpi, while hexoses were relatively lower in older leaves at TPs 0 and 4 dpi, as well as Glc at 14 dpi (Figure S2), resulting in increasing Suc/hexose ratios (data not shown). In addition, from TP −3 dpi to TP 14 dpi, the Suc concentrations increased in all three leaves, while the Glc and Fru concentrations slightly decreased, also resulting in increasing Suc/hexose ratios (Figure S3).

### 2.3. Levan-Type Fructans Directly Inhibit Venturia inaequalis Growth

**Figure 5:**
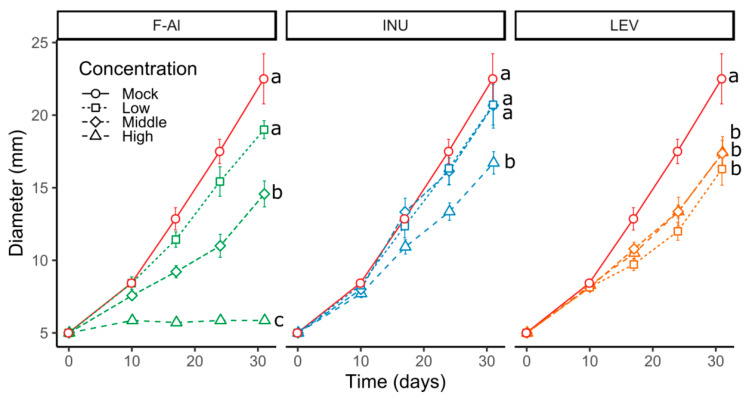
(**A**) Radial mycelial growth of *V. inaequalis* on peptone dextrose agar plates supplemented with fosetyl-Al (F-Al) and fructans (INU = inulin and LEV = levan) at different concentrations (*n* = 7 ± SE) Letters indicated significant differences between the concentrations (*p* < 0.05). The concentrations used are as follows. F-Al: high, 12.5 g L^−1^, medium, 1.25 g L^−1^ and low, 0.125 g L^−1^ and levan and inulin: high, 1 g L^−1^, medium, 0.1 g L^−1^ and low, 0.01 g L^−1^. This experiment was repeated 2 times with consistent results.

Next, we investigated whether fructans have a direct effect on the *V. inaequalis* mycelium development or whether the observed resistance is mediated by fructan effects on the plant. To address this question, we performed mycelial radial growth assays by using a solid growth medium supplemented with F-Al, inulins or levans at three different concentrations (referred as low, medium and high; Figure 5). The mycelial growth was followed for 31 days. As expected, the results obtained for F-Al showed a dose-dependent mycelial growth inhibition (Figure 5). Regarding the fructans, plates supplemented with inulin displayed a reduced mycelial growth (by 22.5% at 31 dpi) compared to the control only for the highest concentration (1 g L^−1^). In contrast, plates supplemented with levans showed a significantly reduced mycelial growth (by 22.5–27.6% at 31 dpi) for all concentrations tested, but without any dose-dependent effect (Figure 5). These data suggest a direct effect of fructans on the *V. inaequalis* mycelium development. 

## 3. Discussion

Increasing costs, development of resistance and environmental concerns urge the development of sustainable and safe alternatives for pesticides [17,18]. The use of biocontrol organisms to counteract apple scab offers a promising alternative or complement to current practices. However, to date, only a limited number of organisms have been properly tested, including the fungi *Cladosporium cladosporioides* and *Athelia bombacina* and the bacteria *Bacillus* spp., *Flavobacterium* sp. and *Pseudomonas syringae* [64,65,66], pointing out the need for additional alternatives. 

In this study, we demonstrated that priming by the exogenous application of levan-type fructans is a promising strategy to further develop a biocontrol method against apple scab caused by *Venturia inaequalis*. Our observations on the “youngest” three apical leaves (with leaf 3 being the oldest among these) showed, for the first time, that the application of levans limits the development of *V. inaequalis in vivo* at a level similar as upon F-Al treatments. Moreover, a direct antifungal effect of levans is demonstrated by fungal growth experiments on agar plates (Figure 5). Interestingly, inulin, but not levan, represents a source of carbon for *V. inaequalis* (Figure 5*;* [67]). Although inulins show a similar antifungal effect to levans at the highest doses *in vitro* (Figure 5), priming with inulin does not provide protection *in vivo*, contrary to levans (Figure 2, Figure 3 and Figure S1). This suggests that levan boosts the intrinsic resistance of apple seedlings to *V. inaequalis* to a much higher extent as compared to inulin, the latter acting as a priming component in the *Botrytis* *cinerea*/lettuce pathosystem [53].

Unfortunately, there are no reports on the perception of levans, neither by higher plants nor by *V. inaequalis*. However, it was suggested before that levans, after the eventual processing by endogenous 6-FEHs (fructan 6-exohydrolases) [68], may be perceived as MAMPs through yet unknown receptors [57,60]. Although the possible downstream processes are unknown, it can be hypothesized that extracellular fructan signaling may be linked to intracellular Suc or Glc signaling. Our sugar analyses revealed no such dynamics (Figure 4), but caution is warranted, since no intermediate TPs between −3 dpi and 0 dpi were considered. Moreover, statistically significant Suc increases were observed in leaf 1 of F-Al-treated plants at 0 dpi. Our recent data on levan oligosaccharide priming in the context of the Arabidopsis/*Botrytis* pathosystem revealed a temporal Suc peak at −2 dpi, possibly indicating a Suc-signaling event (Janse van Rensburg et al., unpublished data). Taken together, based on our data, it cannot be excluded that Suc-signaling effects at the earlier stages after levan priming influence disease resistance, hence urging the need for further research to confirm this hypothesis. 

Taking into account that endogenous sugar levels evolve with leaf ontogeny (Figure S2), overall time (Figure S3) and pathogen inoculation (Figure 4), exogenous fructan treatments were found to affect sugar levels to a lower degree at the post-inoculation stages. In the *B. cinerea/*lettuce pathosystem, the inulin application did not significantly affect the soluble sugar levels at 0 and 1 dpi [57], although the sugar levels dropped in the mock controls [57]. Strikingly, similar dynamics in the sugar levels were also observed in the *B. cinerea*/Arabidopsis pathosystem (Janse van Rensburg et al., unpublished data), indicating that pathosystem-specific effects may influence the leaf sugar levels. In the *Venturia*/apple pathosystem, it is known that *V. inaequalis* uses Glc as one of its preferred carbon sources [69]. However, surprisingly, no significant decreases in Glc levels were recorded in infected leaves as compared to the mock-inoculated ones (Figure 4), with Glc also representing the dominant soluble sugar in apple leaves in the time window considered (Figure 4). Therefore, it is likely that Glc sustains the initial phases of *V. inaequalis* growth without significantly impacting the overall leaf sugar concentrations. This could be due to a replenishing of the leaf Glc pools from the Suc pools and the associated stabilization of leaf Suc levels by differentially regulating the leaf Suc import or export in the different types of leaves. After longer infection times, the Suc levels increase in infected leaves, possibly by further inhibiting the leaf Suc export (Figure 4 and Figure S3). For other pathosystems, it has been demonstrated that Suc accumulation and increased Suc/hexose ratios are associated with Suc-signaling events that activate immune-signaling processes, leading to the synthesis of an array of secondary metabolites, of which many possess antifungal activities [70,71]. However, a high sugar availability can be a double-edge sword for the host, especially when dealing with specific pathogens equipped with extremely efficient sugar importers [72]. The situation is further complicated by time-dependent Suc dynamics as a function of leaf development, which cannot be attributed to *Venturia* inoculation (Figures S2 and S3). 

Besides possible sugar-signaling effects, based on our recent observations in the *B. cinerea*/Arabidopsis pathosystem, we anticipate the putative involvement of heavy crosstalk between sugars, hormones and reactive oxygen species (ROS) signaling. In lettuce, for example, the ethylene signaling and ROS dynamics were shown to be involved in inulin-dependent resistance against *B. cinerea* [57]. Such studies focusing on the involvement and integration of various other endogenous factors offer intriguing perspectives to understand the overall role of fructans during stress tolerance. 

As a further indication of the overall complexity of the pathosystem, we observed little correlation between apple scab sporulation on differentially treated leaves and the macroscopically observed infection symptoms, such as chlorosis and necrosis (Figure 2A, Figure 3 and Figure S1). Such a discrepancy indicates that levan applications may differentially affect disease symptoms or, alternatively, induce an incomplete disease protection under our conditions, hence requiring further investigation. Although current levan-mediated scab protection in apple seedlings provides encouraging data, particularly considering the enhanced protection of older leaves compared to younger ones, future research will have to focus on mature, fruit-bearing apple trees in the context of commercial valorization, as well as include a more rigorous comparison with commercially available fungicides. However, before entering such stages, further optimization of the doses and priming frequency may be warranted at the seedling stage.

## 4. Materials and Methods

### 4.1. Plant and Inoculation Materials

For the experiments, apple seedlings were obtained from a breeding company Better3Fruit (B3F, Rillaar, Belgium). In the priming experiments, seedlings from a “Mariri red” × B3F selection (20/6/42) progeny were used. Based on our previous observations and the breeding experience of B3F, all plants originating from crosses between susceptible parents exhibit a similar susceptibility to *V. inaequalis* and could mostly be classified in Chevalier class 4 as very susceptible (personal communication, B3F; [73]). 

Seedling production started with a minimum of three months of seed stratification, seed germination and potting into soil (DCM Pepi 3, Grobbendonk, Belgium). After potting, seedlings were transported to the greenhouse and grown under controlled greenhouse conditions: a day/night temperature 20/16°C and a day/night relative humidity (RH) of 70%/60%. A photoperiod of at least 16 h of daylight and 8 h of darkness was used with the application of artificial lighting (25 W m^−2^) from high-pressure sodium lamps (400 W Son-Tagro, Philips, The Netherlands) during the day when the light intensity dropped below 100 W m^−2^. For each experiment, plants were grown for approximately six weeks until the state of a minimum of four unfolded leaves. Before the priming and the subsequent inoculation, plants were placed according to a completely randomized design. 

### 4.2. Priming and Infection Assays 

**Figure 6:**
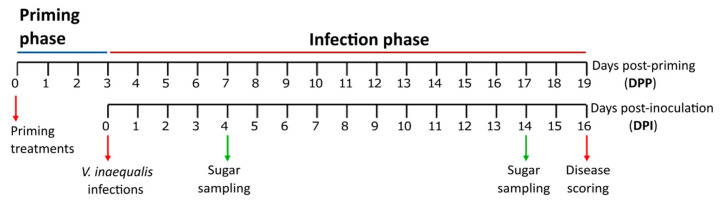
Schematic diagram of the experimental setup used for priming experiments.

In order to perform priming treatments, the three youngest leaves (Figure S5) from 40-day-old apple seedlings were sprayed with the priming solution (see next paragraph—“Preparation of priming agents and solutions”). Our previous findings showed that inulin-type fructans from chicory can reduce disease symptoms in the *B. cinerea/*lettuce pathosystem when sprayed at a concentration of 1 g L^−1^ [53]. Thus, we used the same concentration in our pathosystem. Treatments were applied 3 days before the inoculation, with a 100-mL glass-bottle sprayer. Priming solution was applied on the specified leaves until run-off (approximately 2 mL). Eighteen plants were used per condition tested (TP/treatment/inoculation type). All the solutions were prepared in Milli-Q water supplemented with 0.0001% Tween-20 as surfactant. Negative control (“mock”) consisted in Milli-Q water supplemented with 0.0001% Tween 20. At 3 days post-priming (DPP), plants were inoculated with *V. inaequalis* inoculation solution (see paragraph “Fungal material and artificial inoculation”), incubated in the dark at 100% relative humidity for two days. Disease symptom progression was followed-up until 16 days post-inoculation (dpi) (Figure 2). The disease scoring was performed according to the methodology described below. For the sugar analysis, three leaves at specific positions (age; e.g., leaf 1) were pooled in a single sample (biological replicate, in total six replicates). The samples were collected at the TPs specified in Figure 6. Finally, the samples were immediately frozen in liquid nitrogen and stored at −80 °C until further analysis. 

### 4.3. Preparation of Priming Agents and Solutions

Inulin extracted from chicory roots (*Cichorium intybus*) was purchased as a powder from Sigma-Aldrich (St. Louis, MO, USA). 

For levan preparation, 200 g *Dactylis glomerata* leaves were floating in a 100 mM Suc solution for 2 days in continuous light in order to induce levan accumulation [74]. This material was then washed with water and treated with 800 mL 100% ethanol at 80 °C for 10 min to remove pigments. After filtration, the leaves were extracted with 800 mL water at 85 °C for 15 min. After cooling, this extract was passed through a cheese cloth and adjusted to pH 11 with Ca(OH)_2_ (liming process). The pH of the extract was subsequently lowered to pH 8 by the perfusion of gaseous CO_2_ in the solution (carbonation process). This led to the formation of calcium carbonate (CaCO_3_) precipitates that were removed by centrifugation (8000× *g* for 10 min). The steps of liming and carbonation were repeated twice. The obtained extract was concentrated with a rotary evaporator and passed through a cation and an anion exchange column, composed by two layers of resins (Dowex 50 W × 8 H^+^ and Dowex 1 × 8 100–200 mesh Ac^−^ (Sigma-Aldrich)). The column was washed with 3-column volumes of water. Total flow-through was again concentrated with a rotary evaporator. Levans were finally precipitated by adding acetone to a final concentration of 60%. After washing (two times with 60% acetone), the precipitate was freeze-dried with the use of a lyophilizer (LSL Secfroid, Aclens, Switzerland).

All the obtained powders were weighed, dissolved and analyzed with High-Performance Anion-Exchange Chromatography with Integrated Pulsed Amperometric Detection (HPAEC-IPAD; Dionex 3000, Thermo Fisher, Waltham, MO, USA). A CarboPac^®^ PA100 anion exchange column was used equipped with a gold electrode (potentials: E1: +0.05 V, E2: +0.6 V and E3: −0.8 V). The flow rate was 0.25 mL min^−1^. The column was equilibrated with 90 mM NaOH before injection. Carbohydrates were eluted with a Na-Ac gradient: 0 to 10 mM from 0 to 6 min and 10 to 100 mM from 6 to 45 min. Finally, the column was regenerated with 500 mM Na-Ac for 5 min. Polysaccharides were dissolved in water before use to the concentrations indicated in the text. A comparison between the chromatograms obtained from the purified solutions is shown in Figure S4. F-Al powder was purchased from Bayer CropScience (Leverkusen, Germany) with the commercial name Aliette^®^.

### 4.4. Fungal Material and Artificial Inoculation

To determine the effects of the priming compounds on the resistance of apple to *V. inaequalis* and on the internal sugar levels, all experiments were performed using conidial spores of *V. inaequalis* strain 104, the reference strain of race 1. This strain of race 1 was chosen based on previous experiences with successful and standardized infection experiments with stable symptoms expression in all apple plants without monogenic resistance genes (susceptible plants) [75].

Before the experiment, infected leaves from previous experiments were collected to prepare a conidial spore solution according to Daniëls et al. [76]. In short, leaves with sporulation after infection with the *V. inaequalis* strain 104 were sampled, dried and stored at −20 °C. Subsequently, frozen dry leaves were soaked in cold water (4 °C) for 5 min. *V. inaequalis* spores were filtered from leaf debris, counted and diluted in distilled water to 2 × 10^5^ spores mL^−1^. To standardize the inoculations, later visual evaluations and sampling by leaf position, leaf 1 (the youngest fully unfolded leaf) was labeled as shown in Figure S5. The standard infection conditions and complete leaf wetness were ensured by incubation of the plants in a completely dark infection tunnel with active air humidifiers (Boneco 7135, Widnau, Switzerland) at 100% relative humidity and day-night temperatures of 20–16 °C. Immediately after plants were taken out of the infection tunnel, they were placed under controlled greenhouse conditions, as previously described, with continuous water sprinkling for 15 s every 5 min for 10 consecutive days to ensure sufficient leaf wetness to stimulate the infection process. 

### 4.5. Visual Evaluation Assay of Macroscopic Symptoms

The standardized approach of determination of disease susceptibility/resistance is commonly based on a macroscopic symptoms evaluation [73]. The evaluation is performed at least every second day and is based on a method developed by Croxall et al. [77] and modified by Didelot et al. [78]. In short, the leaf symptoms were evaluated according to three characteristics, i.e., chlorosis, sporulation and necrosis. Sporulation was determined as a percentage of the leaf surface and divided into eight classes (classes 0–7: respectively, 0%, 0–1%, 2–5%, 6–10%, 11–25%, 26–50%, 51–75% and >75%), while the degree of chlorosis and necrosis as a percentage of the leaf surface are divided over five classes (classes 0–4: respectively, 0%, 1–25%, 26–50%, 51–75% and >75%) [76]. The symptoms often start as chlorosis turned into sporulating lesions sometimes accompanied by necrosis covering the chlorosis. Such a transition in visual symptoms can result in chlorosis decrease over time.

All three classifications (chlorosis, sporulation and necrosis) were integrated via the Townsend-Heuberger formula for each plant at each TP. As such, the overall symptom severityer plant [79] was calculated as follows [78]:

(1) $$TH=\frac{\sum n_{i}\cdot i}{N\cdot I}\cdot 100\%.\;$$

where, for each symptom type: *n_i_* = number of leaves in class *i*, *i* = class (0–I), *N* = total number of leaves and *I* = number of symptom-showing classes.

### 4.6. DNA Extraction and Quantification Assay of Venturia DNA

Before DNA extraction, leaves were lyophilized (Freezone 4.5, Labconco, Kansas City, MO, USA), and the whole leaf was ground. Total genomic DNA (gDNA) was extracted from 30-mg lyophilized leaf tissue (from each labeled leaf (leaves 1–3) separately) using the DNeasy Plant Mini Kit (Qiagen, Hilden, Germany) according to the manufacturer’s instructions. DNA concentrations and purities were spectrophotometrically determined (NanoDrop 2000c, Thermo Fisher Scientific, Waltham, MA, USA). Real-time PCR analysis was performed according to the protocol developed by Torfs et al. [80]. Fungal DNA was quantified by means of a Taq-man assay with external fungal standard curves.

### 4.7. Carbohydrate Extraction, Processing and Analysis

To extract and quantify the Glc, Fru and Suc, lyophilized leaf material with a weight of 30 mg was used. Nine-hundred microliters of Milli-Q water was added immediately, followed by boiling for 15 min. After cooling at room temperature, the extract was vortexed and centrifuged at 15,000× *g* for 5 min. Next, 200 µL of the resulting supernatant was loaded on a glass column with two ion exchange resins (200 µL Dowex^®^—50 H^+^ and 200 µL Dowex^®^—1-Ac) and washed six times with 200-µL Milli-Q water. This diluted extract was collected in 1.5 mL Eppendorfs. Ten microliters of this neutralized fraction was then diluted in 90 µL of 20 µM rhamnose (used as the internal standard). The sample was then vortexed and centrifuged at 13,000× *g* for 5 min, transferred to a glass vial with a conical glass insert and analyzed by HPAEC-IPAD to determine the sugar content after separation on a CarboPac^®^ PA100 anion exchange column with pulsed amperometric detection and equipped with a gold electrode (potentials: E1: + 0.05 V, E2: + 0.6 V and E3: − 0.8 V). The flow rate was 0.25 mL min^−1^. The column was equilibrated with 90 mM NaOH before injection. The sugars were eluted with 90 mM NaOH for 10 min. Quantification was performed on the peak areas with the external standards method for Glc, Fru and Suc. External standards consisted of Glc, Fru and Suc at a concentration of 10 µM. Only the peaks exceeding the baseline noise by a factor of 20 were considered.

### 4.8. Media Preparation and In Vitro V. inaequalis Growth on the Plate

Colonies of *V. inaequalis* were isolated from leaves used for inoculation. In short, *V. inaequalis* spores were collected by rubbing sporulation lesions with wet cotton swabs, followed by collecting the spores in demineralized water. Spores were isolated on water agar medium (3% *w*/*v*) until germination (i.e., 24 h). Furthermore, individual spores were transferred to a malt extract agar medium (3.5% *w*/*v*). After the formation of colonies, cylinders measuring 5 mm in diameter were cut and transferred to a fresh medium containing various concentrations of tested compounds (Figure 5). Growth was followed-up by measuring the colony diameters (radial mycelial growth).

### 4.9. Statistical Analysis

Statistical analyses were performed with the computing environment statistical software platform R (version 3.4.3). To determine the significant differences in the non-normally distributed data of the pathogen DNA, the symptoms and sugar content results were subjected to the Wilcoxon pair test for TPs, inoculations, leaf positions and treatments. For mycelial growth analyses, the data were log-transformed, and one-way ANOVA followed by a post-hoc test (Tukey’s honest significant difference (HSD)) were performed.

## 5. Conclusions

A steadily increasing effort from the scientific community is being directed towards developing biological alternatives to pesticide use.

In this study, we show, for the first time, that the exogenous application of levan, a cheap and sustainable compound, can protect the leaves of apple seedlings to a similar extent as F-Al, a synthetic fungicide. Pending further optimization, such application holds great promise to control apple scab diseases.

Further research to better understand the underlying molecular mechanisms of fructan-triggered resistance is warranted—in particular, into the timing of possible sugar-signaling events and the crosstalk with hormonal and ROS signaling.

Such insights may offer novel tools to potentially control many other diseases in an array of agricultural and horticultural crops.

## Supplementary Materials

The following are available online at https://www.mdpi.com/1422-0067/21/16/5885/s1. Figure S1: Necrotic and chlorotic symptoms of *Venturia inaequalis* on apple leaves sprayed with different elicitors. Figure S2: Soluble sugar concentrations in mock-inoculated and mock-treated leaves at three different positions. Figure S3: Soluble sugar concentrations over all four treatments in mock inoculated leaves as a function of time. Figure S4: Comparison of chromatograms of the fructan solutions used as priming agents. Figure S5: Photograph of a representative apple seedling.
