# Supplementary material for: Sweet Immunity: The Effect of Exogenous Fructans on the Susceptibility of Apple (Malus × domestica Borkh.) to Venturia inaequalis
Source: Int J Mol Sci. 2020 Aug 16;21(16):5885. doi: 10.3390/ijms21165885 (PMC7461573; doi:10.3390/ijms21165885)
Supplement: Supplementary file 1 [file ijms-21-05885-s001.pdf]

Supplementary Materials.

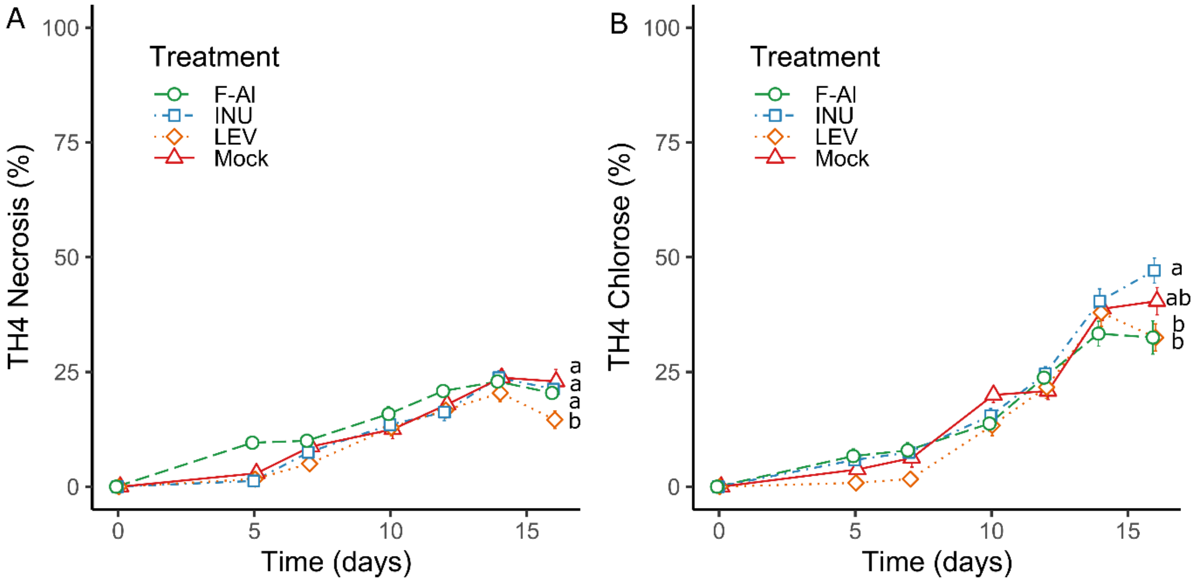

Figure S1. (**A**) Means of TH4 necrotic symptoms of *Venturia inaequalis* on three apple leaves sprayed with different elicitors. F-Al = fosetyl-Al 1.25 g L^{-1}, INU = inulin 1 g L^{-1}, LEV = levan 1 g L^{-1}, Mock = water-treated and TH4 = Townsend-Heuberger with the number of symptom-showing classes (n = 20 ±SE). (**B**) Means of TH4 chlorotic symptoms of *V. inaequalis* on apple leaves sprayed with different elicitors. F-Al = fosetyl-Al 1.25 g L^{-1}, INU = inulin 1 g L^{-1}, LEV = levan 1 g L^{-1} and Mock = water-treated (n = 20 ±SE). Statistical significance at 16 days post-inoculation is indicated by different letters ($p < 0.05$). This experiment was repeated 3 times with consistent results.

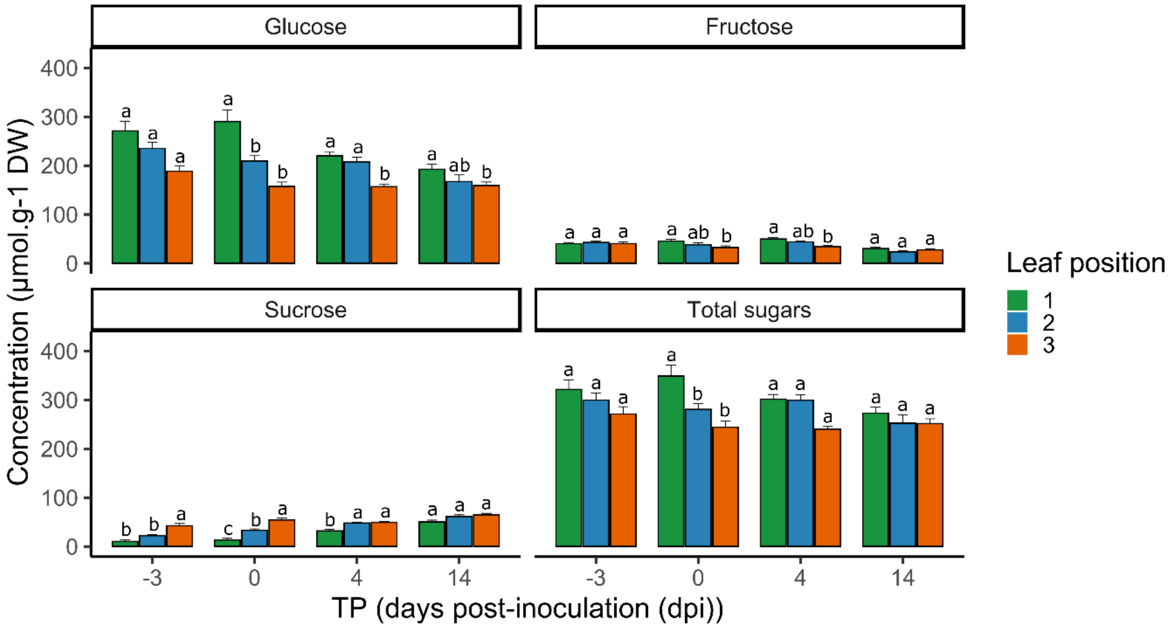

Figure S2. Mean of soluble sugar concentrations (in µmoles/g DW) in mock-inoculated leaves 1, 2 and 3, treated with water. The Y-axis represents the soluble sugar concentration in µmoles/g DW (n = 6 ±SE), while the X-axis represents the sampled time points (TP) in days post-inoculation (dpi). Total sugars are defined as the sum of the glucose, fructose and sucrose concentrations. Statistical significance between the different leaves is indicated by different letters ($p < 0.05$). This experiment was repeated 3 times with consistent results.

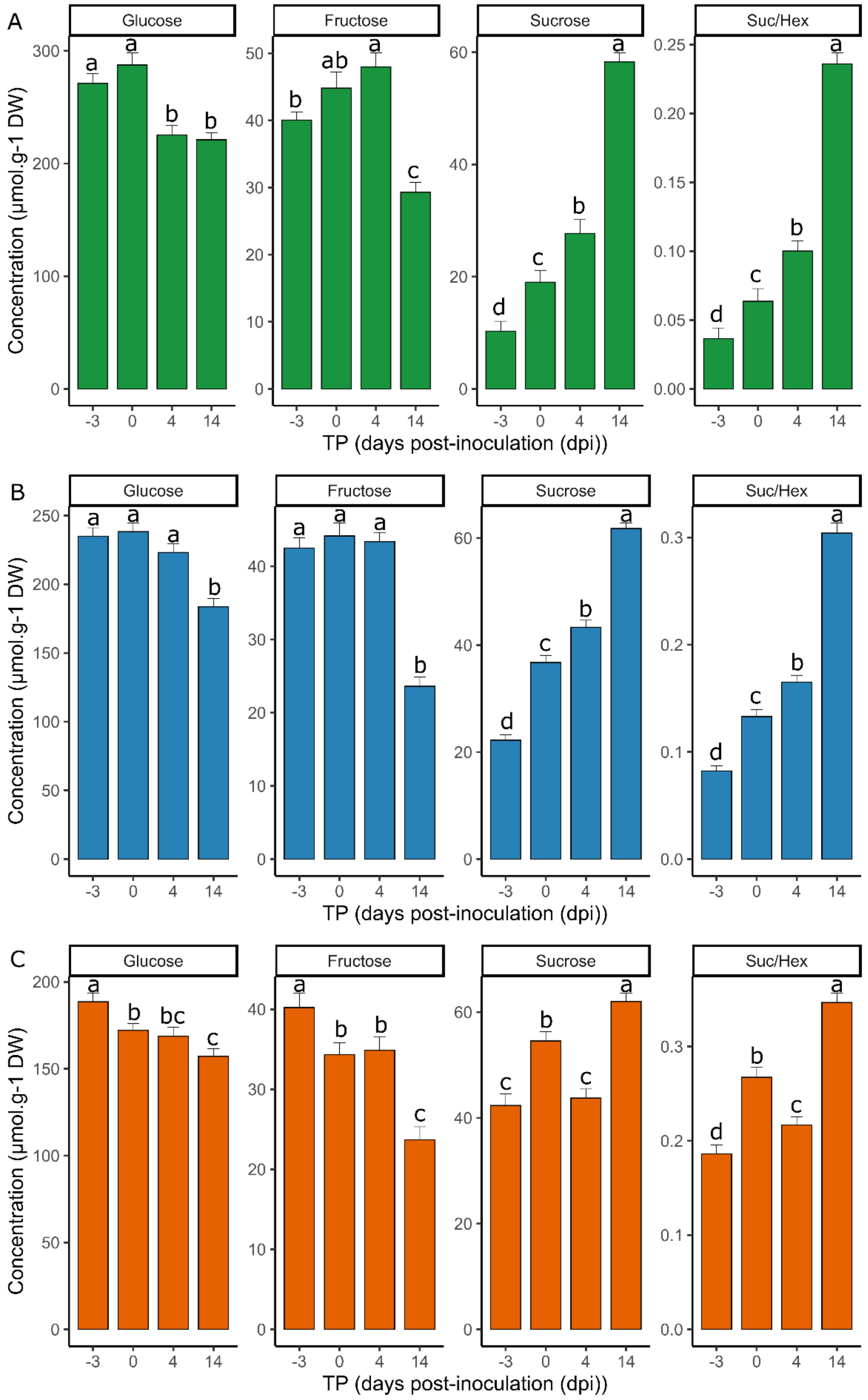

Figure S3. Mean of soluble sugar concentrations (in µmoles/g DW) over all four treatments in mock-inoculated leaves as a function of time (dpi). TP = time point and dpi = days post-inoculation. Total sugars are defined as the sum of the glucose, fructose and sucrose concentrations. (**A**) Sugar concentrations in leaf 1. (**B**) Sugar concentrations in leaf 2. (**C**) Sugar concentrations in leaf 3 (n = 6 ±SE). Letters indicate statistical differences between the TPs ($p < 0.05$).

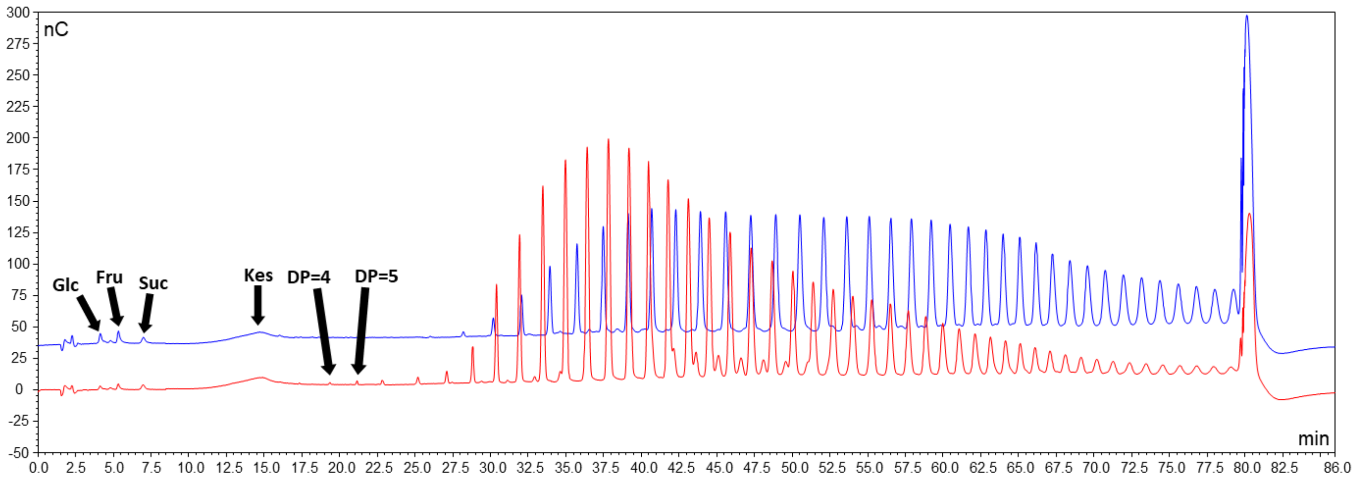

Figure S4. Comparison of chromatograms of the fructan solutions used as priming agents in this study. Blue line = levan and red line = inulin. Glc = glucose, Fru = fructose, Suc = sucrose and Kes = 1- kestose. Subsequent peaks represent fructans with a higher degree of polymerization (DP). Y-axis = detector response in nanoCoulomb (nC) and X-axis = retention time in min.

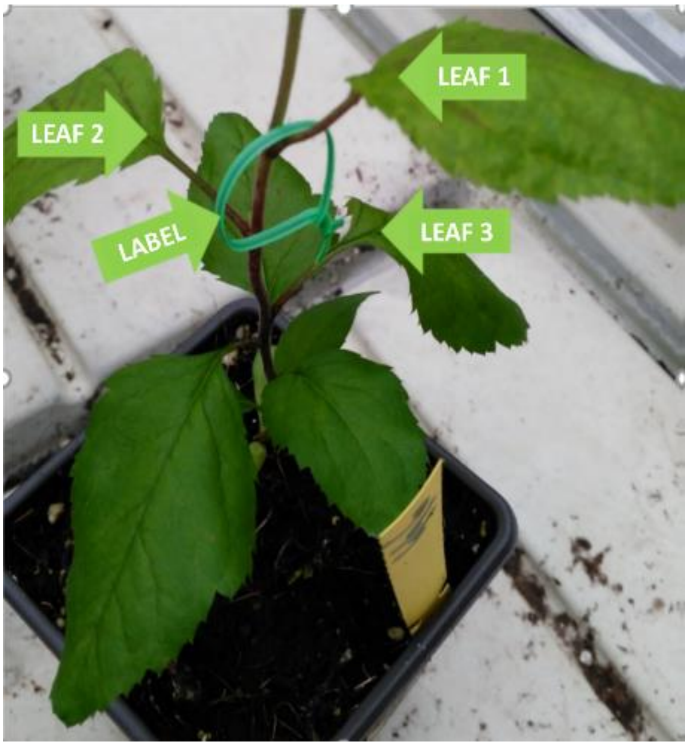

Figure S5. Photograph of a representative apple seedling used in this work, showing the positioning of the leaves relative to the apex used for priming, sampling and disease scoring assays.
